# Editorial: Immune metabolism in auto- and allo-immunity

**DOI:** 10.3389/fimmu.2024.1483411

**Published:** 2024-09-25

**Authors:** Lalit Basur, Haitian Zhou, Jolin Cheng, Yinuo Zang, Guiyu Song, Liying He, Yulong Lan, Zihan Ma, Alain Haziot, Dan Jane-wit

**Affiliations:** ^1^ School of Clinical Medicine, University of Cambridge, Cambridge, United Kingdom; ^2^ Section of Cardiovascular Medicine, Yale University School of Medicine, New Haven, CT, United States; ^3^ Department of Cardiology, West Haven VA Medical Center, West Haven, CT, United States; ^4^ Department of Obstetrics and Gynecology, Shengjing Hospital of China Medical University, Shenyang, China; ^5^ Department of Neurosurgery, Second Affiliated Hospital, School of Medicine, Zhejiang University, Hangzhou, Zhejiang, China; ^6^ Institut National de la Santé et de la Recherche Médicale (INSERM) U976, Paris, France; ^7^ Université de Paris, Institut de Recherche Saint Louis, Paris, France

**Keywords:** immune metabolism, systemic lupus erythematosus, type 1 diabetes, transplant rejection, alloimmunity, autoimmunity

The field of immune metabolism has garnered burgeoning interest among the scientific community. Diverse cellular processes related to immune cell activation (1) as well as tolerogenic programs (2) are regulated by metabolic pathways, supporting a global assertion that metabolic changes fundamentally shape the immune response. The critical link between metabolism and immunity is highlighted in the set of research articles collected under a recent Research Topic, ‘*Immune Metabolism in Auto- and Allo-immmunity*,’ appearing in Frontiers in Immunology. This Research Topic consists of seven articles that explore facets of immune metabolism related to autoimmune disease and transplant immunology. We summarize salient findings discussed in these articles below.

A subset of articles appearing in our Research Topic explored immune metabolism in autoimmune disease. Goetz et al. explored the metabolic dynamics of T cells in the context of Systemic Lupus Erythematosus (SLE), an autoimmune condition characterized by systemic inflammation and multi-system organ involvement. Over two decades, significant advancements have been made in understanding the metabolic rewiring of T cells in SLE. However, many of these studies have looked at metabolic pathways in isolation. While previously studied in isolation, a review by Goetz et al. advocates for a systems approach, emphasizing networks inclusive of biomass composition, cytokine secretion rates, and nutrient uptake/excretion rates. This proposed approach, integrating cell physiology experiments, orthogonal metabolic measurements, and computational modelling is intended to uncover insights into pathogenic effects of T cells in SLE that have been overlooked using present-day, traditional approaches. The authors believe that holistic understanding of T cell metabolism will lead to new therapeutic strategies for SLE.

Lipid and glucose metabolism form a bifurcating intersection with implications for fate decisions in immune cells. A second article by Zhang et al. reviewed the intricate role of lipid metabolism in the onset and progression of Type 1 Diabetes Mellitus (T1DM). In this review, Zhang et al. discusses the contribution of various lipids including fatty acids, sphingolipids, glycerolipids, and sterol lipids in the inflammatory responses associated with T1DM. The review emphasizes the potential of lipid profiles as biomarkers for early detection and the possibility of lipid-based therapeutic interventions. Moreover, it highlights the necessity for further research to understand the molecular mechanisms of lipids in immune-mediated β-cell destruction and the benefits of targeted nutritional strategies in preventing and managing T1DM.

A complementary review in this Research Topic by Kemp et al. provides a description of fatty acid oxidation (FAO) and how this metabolic pathway affects various immune cell subsets. The authors provide a comprehensive review of the role of FAO across cells mediating innate immunity including innate lymphoid cells (ILCs), dendritic cells, and macrophages as well as cells mediating adaptive immunity including B cells, T cells, and natural regulatory T cells (Tregs). A broad range of immune settings implicating changes in FAO spanning from infection, cancer, and transplantation are discussed in this Review.

In a Research article, Kong et al. examined the metabolic effects of narcilasine (NCS), a plant alkaloid, in the setting of psoriasis, an autoimmune skin condition characterized by debilitating skin inflammation. NCS is responsible for various types of biological activities related to both immunity and metabolism, and the authors of this paper have examined the ability of NCS to block psoriasis-like dermatitis. The authors demonstrate that NCS attenuated psoriatic skin lesions and epidermal thickening, reducing immune cell infiltrates and inflammatory mediators. These changes were accompanied by decreased proliferation, a phenotype linked to altered metabolism. To interrogate these responses, the authors combined transcriptomic and lipidomic analyses and identified a key role phospholipid metabolism to explain the anti-inflammatory effects of NCS. The phospholipase A2 family genes have a role in regulating inflammation and skin homeostasis and were strongly downregulated in mouse skin by NCS treatment. Decreased phospholipase A2 subsequently led to decreased pro-inflammatory lipid mediators. Single-cell RNA sequencing further identified keratinocytes as key target cells for the metabolic actions of NCS. The author state that NCS could become a very key therapeutic candidate for the treatment of psoriasis and possibly other inflammatory skin diseases.

In a Research article, Li et al. explored the role of proline metabolism in asthma, a highly prevalent condition characterized by airway hyperreactivity. Using a model of Pneumonia Virus of Mice (PVM), a murine equivalent for human respiratory syncytial virus (RSV), the authors demonstrate that macrophages showed features of trained immunity following allergen sensitization. Sensitized macrophages demonstrated gene signatures consistent with increased proline biosynthesis, and inhibition of proline biosynthetic pathways suppressed trained immunity, resulting in decreased allergen sensitivity. These studies demonstrated a link between proline metabolism and allergen sensitivity, indicating a novel means to potentially prevent allergic asthma of childhood.

A second subset of studies focused on immune metabolism in transplant rejection.

In a Perspective article, Burke et al. discussed the value of studying perturbed metabolism following organ transplantation to understand the pathogenesis of autoimmune disease. The authors cite specific cases where certain autoinflammatory develop in the setting of the post-transplant immunologic milieu. One such condition including autoimmune focal segmental glomerulosclerosis (FSGS), where proteinuric renal failure and autoimmune kidney injury develops following kidney transplantation. Other examples cited by the authors include autoimmune pancreatitis following pancreatic islet transplantation, and recurrence of non-alcoholic steatohepatitis following orthotopic liver transplantation. By studying these phenomena, the authors posit that researchers may uncover novel mechanisms and immunologic pathways related to loss of tolerance. These novel insights and therapeutic approaches are relevant not only for post-transplant recurrence but also for development of the original diseases themselves.

FOXP3 is a transcription factor necessary and sufficient for development of regulatory T cells (3, 4) and whose gene variants lead to polyglandular autoimmunity in a condition termed IPEX (5). In a Research article, Saleh et al. examined the relationship between the full-length splice variant of the FOXP3 gene and kidney allograft tolerance. The study highlights that the expression of this specific splice variant is significantly associated with patients who exhibit long-term tolerance to kidney transplants without the need for continuous immunosuppression. Through a combination of gene expression analysis and clinical data, the authors propose that the presence of this variant can serve as a potential biomarker for identifying patients more likely to achieve transplant tolerance, thus advancing personalized treatment strategies in transplant immunology.

In conclusion, articles in this Research Topic highlight important aspects of immune metabolism that are broadly relevant to auto- and allo-immunity. These articles may additionally serve as a relevant reference as to future conduct of research into this field.
